# Acute Phase Extrapulmonary Effects of a High-Dose Influenza A Virus Infection in a Mouse Model of Obesity

**DOI:** 10.3390/zoonoticdis5040031

**Published:** 2025-10-16

**Authors:** Saranya Vijayakumar, Saurav Pantha, Brian Wolfe, Qi Zhang, Shristy Budha Magar, Tawfik Aboellail, Santosh Dhakal

**Affiliations:** 1 Department of Diagnostic Medicine/Pathobiology, College of Veterinary Medicine, Kansas State University, 1800 Denison Avenue, Manhattan, KS 66506, USA; 2 Kansas State Veterinary Diagnostic Laboratory (KSVDL), College of Veterinary Medicine, Kansas State University, 1800 Denison Avenue, Manhattan, KS 66506, USA

**Keywords:** influenza A virus, obesity, extrapulmonary pathology, cytokines, chemokines, systemic inflammation

## Abstract

Influenza A viruses (IAVs) primarily cause respiratory illness but can also induce extrapulmonary effects, which may be aggravated by obesity. This study evaluated the impact of obesity on virus replication, histopathological changes, and cytokine/chemokine profiles in extrapulmonary sites during the acute phase, following a high-dose IAV infection. Diet-induced non-obese mice or mice with obesity were inoculated intranasally with either vehicle (medium) or 10^3^ TCID_50_ of the 2009 pandemic H1N1 IAV. At 3 days post-infection (dpi), the lungs, blood, and various extrapulmonary tissues were collected for virus titration, histopathological analysis, and cytokine/chemokine quantification. IAV infection resulted in comparable virus titers (6–7 log_10_ TCID_50_/mL) and histopathological scores (*p* > 0.05 in each case) in the lungs of mice with or without obesity. Replicating viruses were not detected in the extrapulmonary sites, and histopathological scores did not differ significantly between the two groups. However, analysis of fold changes in five cytokines/chemokines (i.e., IL-6, IL-1β, TNFα, MCP-1, and IFNγ) revealed site-specific differences. IL-6 was significantly higher (*p* < 0.05) in the lungs and perirenal adipose tissue, and showed a higher trend in the kidney (0.05 ≤ *p* ≤ 0.1); IL-1β had a higher trend in the lungs; TNFα was significantly lower in the kidney but showed a higher trend in the lungs; while MCP-1 was significantly lower in the lungs, plasma, and inguinal adipose tissue of mice with obesity compared to non-obese mice. Future studies should consider a broader range of IAV strains/subtypes, doses, time points, and inflammatory markers to better understand how obesity affects extrapulmonary outcomes.

## Simple Summary

Influenza A virus (IAV) infection is a significant global public health concern, and obesity is an independent risk factor for severe outcomes of IAV infection. Although IAV primarily replicates in the respiratory tract, severe disease can aggravate systemic inflammation with poorly characterized extrapulmonary effects in obesity. We compared viral titers, histologic inflammation scores, and cytokine/chemokine profiles in the lungs, blood, and extrapulmonary tissues, at the early-state (acute phase), in vehicle (medium)-inoculated and a high-dose IAV-infected mice with or without obesity. Obesity did not alter virus replication and histopathological changes in extrapulmonary tissues during the acute phase. However, cytokine/chemokine responses were altered in extrapulmonary sites in a tissue-specific manner. These findings suggest a mild impact of obesity on amplification of a high-dose IAV infection-induced inflammation in the extrapulmonary tissues during the acute phase.

## 1. Introduction

The influenza virus is a zoonotic pathogen that can transmit from animals to humans, posing a threat in the form of epidemics and pandemics, as seen in the 2009 H1N1 influenza A virus (IAV) pandemic [1]. A recent outbreak of H5N1 IAV in US dairy cattle and documented human cases associated with the bovine strains further highlight the zoonotic significance of IAVs [2]. Influenza virus infection remains a significant global public health concern, contributing to an estimated 3–5 million cases of severe illnesses and 290,000–650,000 respiratory-related deaths each year [3]. Populations at an increased risk for severe disease or complications following influenza virus infection include pregnant women, young children (<5 years of age), older adults, immunocompromised persons, and people with chronic medical conditions [3]. Obesity (i.e., having a body mass index (BMI) ≥ 30) is characterized by a state of low-grade chronic inflammation and is an independent risk factor for increased disease severity and mortality during influenza virus infection, and is associated with inferior protection following influenza vaccination [4-6]. Human and mouse model studies suggest that obesity is associated with increased influenza viral shedding and delayed virus clearance, progression to viral pneumonia, and a higher risk of secondary bacterial infections [7].

IAV infection primarily causes respiratory illnesses, associated with the production of inflammatory cytokines and damage to the respiratory epithelium [8]. Circulating proinflammatory cytokines are also responsible for systemic symptoms such as fever, chills, body aches, and fatigue. Although influenza virus replication and pathological changes primarily occur in the respiratory tract, severe IAV infections are associated with extra-pulmonary complications, including viral myocarditis and viral encephalitis [9,10]. Such extrapulmonary effects are observed more in high-risk groups such as young children, the elderly, and individuals with chronic medical conditions.

In individuals with obesity, visceral adipocytes and infiltrating macrophages in expanding adipose tissues secrete elevated levels of cytokines and chemokines, which trigger systemic inflammation and could contribute to damage during viral infections beyond the respiratory tract [11,12]. Prior studies suggest that adipose tissues may serve as a reservoir for IAV, and obesity-associated adiposity and chronic low-grade inflammation play important roles in mediating inflammatory responses systemically beyond the lungs [13-20]. These studies differ in terms of virus strains/subtypes, doses, time points, animal models used, and consistency in tissue types analyzed. Therefore, in this study, we used the C57BL/6J mouse model of diet-induced obesity (DIO) to investigate whether the extrapulmonary effects of a high-dose 2009 pandemic H1N1 IAV infection during the acute phase of the disease are enhanced by the obesity status or not. Our objective was to compare virus replication, histopathological changes, and key cytokine/chemokine alterations in the various extrapulmonary tissues of mice with or without obesity, following a high dose of the 2009 pandemic H1N1 IAV infection at 3 days post-infection (dpi). Comparisons were made, specifically, in the lungs, plasma, adipose tissues (gonadal, inguinal, and perirenal), heart, kidney, spleen, and liver.

## 2. Materials and Methods

### 2.1. Animals

Four- to five-week-old C57BL/6J (strain 000664) male and female mice were obtained from the Jackson Laboratory (Bar Harbor, ME, USA). After a week of acclimatization, the mice were randomly assigned to either a low-fat diet (LFD) comprising 10% kCal from fat sources (D12450J, Research Diets, New Brunswick, NJ, USA) or a high-fat diet (HFD) consisting of 60% kCal from fat sources (D12492i, Research Diets, New Brunswick, NJ, USA) for the remainder of the experiment. The mice were group-housed, with no more than five per cage, following standard animal biosafety level (ABSL)-1 conditions during the diet treatments and under standard ABSL-2 conditions for the infection experiments, with ad libitum access to food and water [21]. The diet was replaced twice a week, and body mass was measured every week. After the 14th week of diet treatment, the body mass of each mouse on HFD was compared with that of the mice on LFD, and obesity was defined as a body mass ≥ 20% greater than the average body mass of age- and sex-matched mice on LFD. Mice that did not obtain ≥20% body mass were referred to as non-responders and are not used in this analysis. We had 20 mice (10/sex) for both LFD and HFD treatments, and those that did not confirm obesity status or had any other health issues were eliminated, resulting in 18 non-obese (8 males and 10 females) and 16 with obesity (9 males and 7 females). The BMI (gm/cm^2^) was determined by dividing the body mass by the square of nose-to-anus length measured after the 14th week of diet treatment. All the animal procedures were approved through the Institutional Animal Care and Use Committee (IACUC) of Kansas State University (protocol #4855).

### 2.2. Glucose Tolerance Test (GTT)

Blood glucose levels in mice were measured in the 14th week from the tip of the tail after a quick prick with a sterile lancet, using the AlphaTrak3 blood glucose monitoring system (Zoetis, Parsippany, NJ, USA). To perform the GTT, mice were transferred to a new cage supplied with water but not food and were fasted for 6 h. After the fast, the water supply was discontinued, and a 25% glucose solution, prepared by mixing D-glucose (Sigma Aldrich, St. Louis, MA, USA) in sterile 1× phosphate-buffered saline (PBS), was administered via the intraperitoneal route at a dose rate of 2 g/kg body mass. Blood glucose measurements were taken at different time points, including before fasting (i.e., to measure the fed glucose), at 0 min (i.e., after fasting and immediately before glucose administration to measure fasting glucose), and at 15, 30, 60, and 120 min after glucose administration. Readings that exceeded the detectable threshold were assigned the maximum detectable value (i.e., 750 mg/dL) for statistical comparisons.

### 2.3. Measurement of Serum Leptin and Total Cholesterol

Plasma samples were obtained by centrifuging blood collected 14 weeks after diet treatment at 2000 rpm for 20 min at 4 °C. The separated plasma was stored at −80 °C until analysis. Plasma leptin and total cholesterol levels were measured using a Mouse Leptin ELISA Kit (Crystal Chem Inc., Elk Grove Village, IL, USA) and Total Cholesterol Assay Kit Colorimetric (Cell Biolabs Inc., San Diego, CA, USA), following the manufacturer’s instructions.

### 2.4. Mouse Infection with the Influenza Virus

After 14 weeks on their respective diets, both male and female non-obese and obese mice were anesthetized with a cocktail of ketamine and xylazine and inoculated intranasally with either Dulbecco’s Modified Eagle Medium (DMEM, Thermo Fisher Scientific, Waltham, MA, USA) or 10^3^ tissue-culture infectious dose 50 (TCID_50_) of mouse-adapted A/California/04/2009 H1N1 IAV diluted in DMEM [21]. In non-obese adult mice, 10^3^ TCID_50_ of the 2009 pandemic IAV represents a dose higher than the mLD_50_, and hence this dose is referred to as a high dose infection [21]. Viruses were kindly provided by Drs. Andrew S. Pekosz and Sabra L. Klein of the Johns Hopkins University and used in earlier mouse infection and vaccination studies [21-23]. Viruses were propagated on Madin-Darby Canine Kidney (MDCK) cells. From the non-obese group, 7 were inoculated with medium and 11 were inoculated with IAV, while from the obese group, 7 were inoculated with medium and 9 with IAV. All the mice were euthanized at 3 dpi, and plasma samples, along with the lungs and other tissues such as the spleen, kidney, heart, liver, gonadal adipose tissue (GAT) (epididymal adipose tissue from male and periovarian adipose tissue from the female), inguinal adipose tissue (IAT), and peri-renal adipose tissue (PAT), were collected. During euthanasia, weights of GAT, IAT, and PAT were also measured.

### 2.5. Quantitation of Virus Titers

Lungs and other tissues collected at 3 dpi were flash-frozen in dry ice, followed by storage at −80 °C. After thawing, the tissues were weighed and homogenized as previously described [21]. The collected supernatants were then aliquoted and stored at −80 °C. The infectious virus titers in the lung and other tissue homogenates were determined by using the MDCK cell-based TCID_50_ assay [24].

### 2.6. Histopathology

At necropsy, the left lung lobes, heart, kidney, liver, and gonadal adipose tissue were collected and immersion-fixed in zinc-buffered formalin (Z-Fix; Anatech, Battle Creek, MI, USA) for at least 72 h to ensure complete virus inactivation [25]. Samples were then submitted to the Kansas State Veterinary Diagnostic Laboratory (KSVDL) for routine trimming and processing. Tissue sections were cut at 5 μm thickness and stained with hematoxylin and eosin (H&E) according to standard histological protocols. Lung sections included four transverse cuts from the left lung. The heart was sectioned midsagittal to include all four chambers. Kidney sections were taken through the hilus, liver sections near the hilus, and the entire GAT was mounted. A board-certified veterinary pathologist, blinded to experimental groups, performed a semi-quantitative assessment of inflammatory and degenerative changes in each tissue, but intraobserver variability was not assessed. Scoring was based on a scale from 0 (absent) to 5 (severe). For the lungs (maximum score = 40), the composite inflammation score included pleural inflammation; airway inflammation in the main stem bronchus, primary bronchiole, and right bronchus; parenchymal inflammation; and vascular inflammation around arteries, veins, and capillaries. For gonadal adipose tissue (maximum score = 25), the average diameter of the eight largest adipocytes within a defined region of interest (ROI) was measured, and semi-quantitative scores were assigned for inflammation, necrosis, calcification, septal fibrosis, and atrophy. Kidney pathology (maximum score = 25) was assessed in the renal capsule, glomeruli, tubules, vessels, and pelvis. Liver evaluation (maximum score = 35) included scoring of capsular, portal, parenchymal, and vascular inflammation, as well as necrosis, degeneration, and hepatocellular proliferation. Cardiac pathology (maximum score = 20) included assessment of the pericardium, left ventricle, right ventricle, and interventricular septum.

### 2.7. Multiplex Cytokine and Chemokine Analysis in Lung Homogenates and Plasma Samples

Cytokines and chemokines in lung homogenates and plasma samples were measured using the ProcartaPlex mouse cytokine and chemokine panel 1, 26plex (#EPX260-26088-901, Thermo Fisher Scientific, Waltham, MA, USA) as per the manufacturer ‘s instructions [21].

### 2.8. Quantitative Reverse Transcription PCR (qRT-PCR) for Cytokines/Chemokines

RNA was extracted from the spleen, kidney, liver, GAT, IAT, and PAT collected at 3 dpi using TRIzol^™^ Plus PureLink RNA mini kit (#12183555, Thermo Fischer Scientific, Waltham, MA, USA). The purity and concentration of RNA were assessed using a NanoDrop 1000 spectrophotometer (Thermo Scientific, Waltham, MA, USA). Complementary DNA (cDNA) synthesis was performed using the High-Capacity cDNA Reverse Transcription Kit (#4368814, Thermo Fisher Scientific, Waltham, MA, USA). mRNA levels of five cytokine/chemokine genes of interest, including Interleukin (IL)-6, Interferon-γ (IFNγ), Interleukin (IL)-1β, Tumor necrosis factor-α (TNFα), and monocyte chemoattractant protein-1 (MCP-1), and housekeeping gene β-actin were measured using the primer sequences as described previously [26-28]. qRT-PCR was carried out on a CFX96 Touch Real-Time PCR instrument (Bio-Rad) under the following thermal cycling conditions: initial enzyme activation at 95 °C for 15 min, followed by 40 cycles of denaturation at 95 °C for 15 s, and gene-specific annealing temperature at 55.3 °C for 60 s for IL-6, IFN-γ, IL-1β, and TNF-α, and 52.1 °C for 60 s for MCP-1, β-actin. All runs included a post-PCR melt curve analysis consisting of 95 °C for 15 s, 60 °C for 1 min, and 95 °C for 15 s. The target gene expression results were subsequently expressed as fold change relative to the medium-inoculated control groups by using the comparative 2^−ΔΔCt^ method [29]. If any samples had poor RNA quality or if Ct value was not detected at all for any cytokines/chemokines in any tissues, that animal data was excluded from analysis.

### 2.9. Statistical Analysis

Statistical analysis was performed, and manuscript figures were prepared in GraphPad Prism 10.5.0 and Microsoft Excel for Microsoft 365 (version 2507). The unpaired *t*-test was used to compare two groups, and two-way ANOVA followed by Tukey’s multiple comparisons was used for statistical comparisons between non-obese and obese, medium-inoculated versus virus-infected mice. GTT data over time were compared using two-way repeated measures ANOVA followed by Tukey’s multiple comparisons. Log_10_-transformed cytokine/chemokine concentrations from 26-plex assay were compared using unpaired *t*-test with Holm–Sidak correction for multiple comparisons. Statistical details are also provided in each figure legend. Statistical significance was defined as *p* < 0.05, and a trend was defined if 0.05 ≤ *p* ≤ 0.1.

## 3. Results

### 3.1. As Expected, the High-Fat Diet (HFD) Treatment for a 14-Week Duration Induced Obesity in C57BL/6J Mice

**Figure 1.**
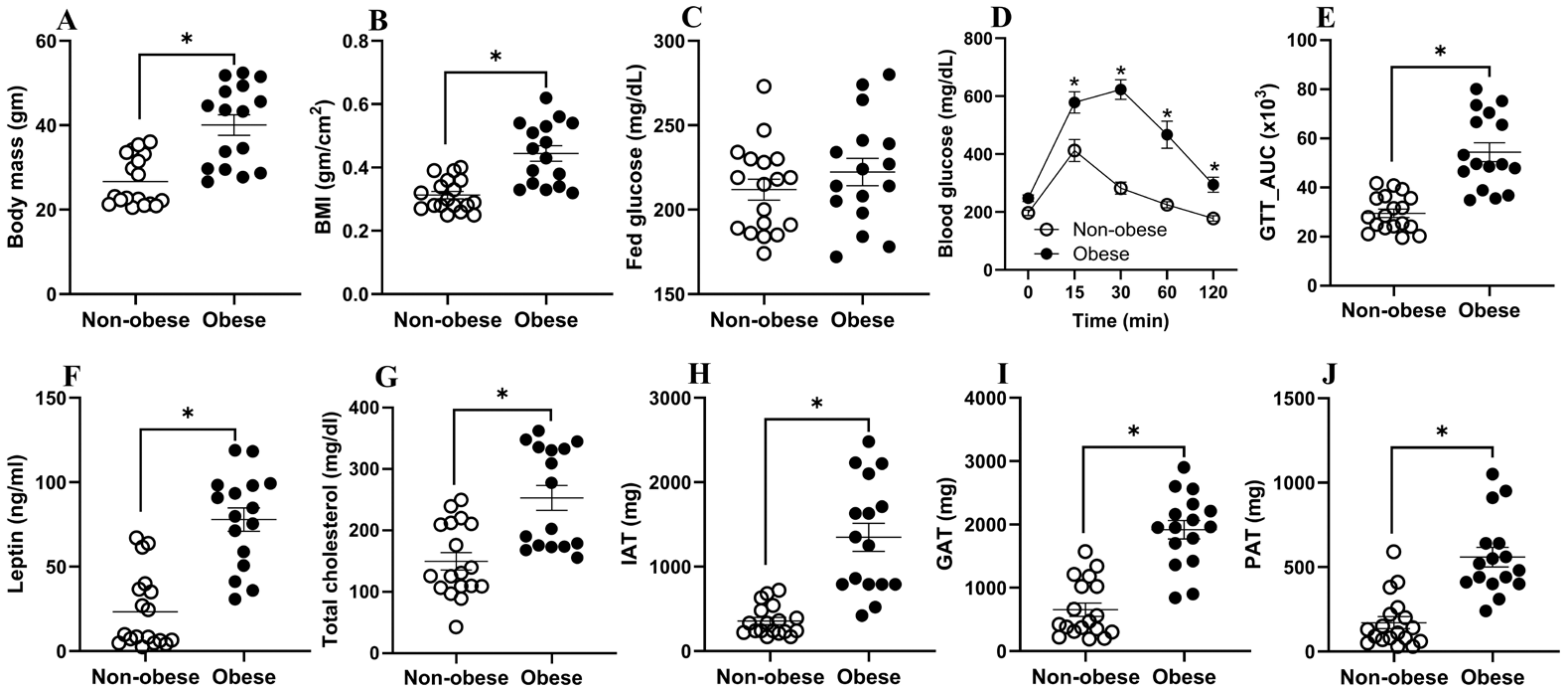
Differences between mice, with or without obesity, following diet treatment. C57BL/6J mice (4–6 weeks old) were treated with a low-fat diet (LFD) or high-fat diet (HFD) for 14 weeks. Body mass (**A**), body mass index (BMI, **B**), fed glucose (**C**), blood glucose over time after glucose tolerance test (GTT, **D**), GTT area under the curve (AUC, **E**), plasma leptin concentration (**F**), and total cholesterol concentration (**G**) were measured at the 14th week. Mice were infected with medium only or IAV and euthanized at 3 days post-infection (dpi), and the weights of inguinal adipose tissue (IAT, **H**), gonadal adipose tissue (GAT, **I**), and perirenal adipose tissue (PAT, **J**) were compared between non-obese and obese mice. Data represent mean ± standard error of mean (SEM) of 16–18 mice/group. Statistical comparison was carried out using an unpaired *t*-test and the asterisk represents significant difference (*p* < 0.05).

Male and female C57BL/6J mice were treated with the LFD or HFD for 14 weeks. As expected, after the 14th week, HFD-fed mice exhibited significantly greater body mass, higher BMI, impaired glucose tolerance as shown by the excursion of blood glucose for a longer duration, and elevated plasma leptin and total cholesterol levels (Figure 1A-G). There was no difference in adiponectin levels. We also observed markedly increased adiposity in the gonadal, inguinal, and perirenal fat (Figure 1H-J), confirming obesity induction as in previous reports of diet-induced obesity [30-32].

### 3.2. High-Dose IAV Infection Caused Inflammatory Changes in the Lungs of Both Groups

**Figure 2.**
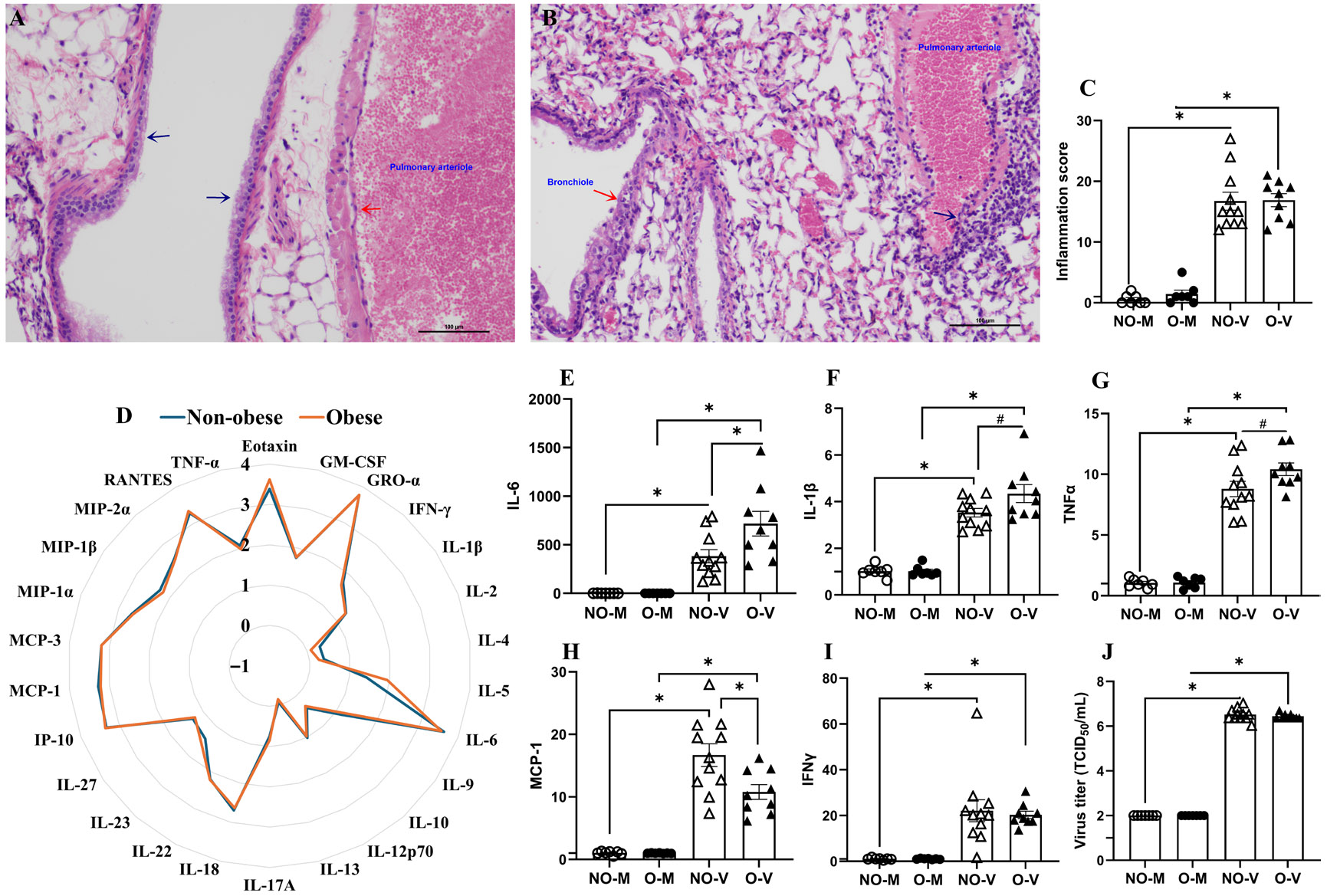
Inflammatory changes and virus replication in the lungs. At 14 weeks after the diet treatment, mice with or without obesity were either inoculated with medium only or a 10^3^ TCID_50_ of 2009 H1N1 IAV. Mice were euthanized at 3 days post-infection (dpi). Left lung lobes were formalin-fixed and stained with hematoxylin and eosin (**E**,**H**) for histopathological analysis. Representative lung images of medium-inoculated (**A**) and virus-inoculated (**B**) female mice with obesity are shown, and lung inflammation scores are compared (**C**). At 3 dpi, 26 cytokines and chemokines were measured in the lungs of medium- and virus-inoculated mice by multiplex ELISA, and log_10_-transformed absolute concentrations of cytokines/chemokines in virus-infected mice with or without obesity are compared (**D**). Likewise, the fold changes in IL-6, IL-1β, TNFα, MCP-1, and IFNγ are shown (**E**–**I**) and were selected for analysis in extrapulmonary tissues. At 3 dpi, replicating virus titers were measured in the lung homogenates using TCID_50_ assay (**J**). Data represent mean ± standard error of mean (SEM) of 7–11 mice/group. Statistical comparison was carried out using the unpaired *t*-test with Holm–Sidak correction for multiple comparisons (**D**) or two-way ANOVA followed by Tukey’s multiple comparisons (**C**,**E**–**J**). The asterisk represents a significant difference (*p* < 0.05), and # represents a trend (0.05 ≤ *p* ≤ 0.1). NO-M, O-M, NO-V, and O-V represent the non-obese medium-inoculated, obese medium-inoculated, non-obese virus-infected, and obese virus-infected groups, respectively.

C57BL/6J mice, with or without obesity, obtained after the LFD or HFD diet treatment for a 14-week duration, were inoculated intranasally either with vehicle (i.e., medium only) or with a high-dose of the 2009 H1N1 IAV and euthanized at 3 dpi. To determine the pulmonary pathological changes, H&E staining was performed in the lung tissues. A representative image of the H&E-stained lungs from medium-inoculated female mice with obesity is presented, which shows a total lack of inflammation in the airways that are lined by pseudostratified ciliated epithelium (blue arrow) and pulmonary artery lined by normal endothelium (red arrow) (Figure 2A). Likewise, a representative image of the H&E-stained lungs from the virus-infected female mice with obesity is presented that shows pulmonary lesions depicting deciliation, focal necrosis, and inflammation of the bronchiolar epithelium (red arrow) and inflammation extending into the wall of the pulmonary arteriole (blue arrow, Figure 2B). Virus-infected mice of both groups had significantly higher (*p* < 0.05) pathological changes compared to their respective medium-inoculated controls. However, there was no significant difference in histopathology scores between the virus-infected mice, regardless of whether they had obesity (Figure 2C).

We also measured 26 different cytokines and chemokines in the lungs of medium and IAV-inoculated mice at 3 dpi. The log_10_-transformed absolute concentrations of the various cytokines/chemokines in the virus-infected mice as compared to their medium-inoculated controls were determined and compared (Figure 2D). There was no difference in the concentrations of cytokines/chemokines in the lung homogenates between the two groups at 3 dpi (Figure 2D). For comparisons of inflammatory changes in other extrapulmonary tissues, proinflammatory cytokines IL-6, IL-1β, TNFα, and chemokine MCP-1, as well as Th-1 cytokine IFNγ, were selected, because in the lungs all these were significantly upregulated in virus-infected mice compared to medium controls (Figure 2E-I).

Replicating virus titers were determined in the lungs at 3 dpi by cell-based TCID_50_ assay. As expected, medium inoculated mice had no virus detected in the lungs, while virus-infected mice had virus titers between 6 to 7 log_10_ TCID_50_/mL, and there was no significant difference (*p* > 0.05) in virus titers in the lungs of both groups of mice (Figure 2J). These data indicate that high-dose IAV infection induces inflammatory changes in the lungs of both non-obese mice and mice with obesity.

### 3.3. Cytokines/Chemokine Alterations in Plasma After IAV Infection Between Mice with or Without Obesity

**Figure 3.**
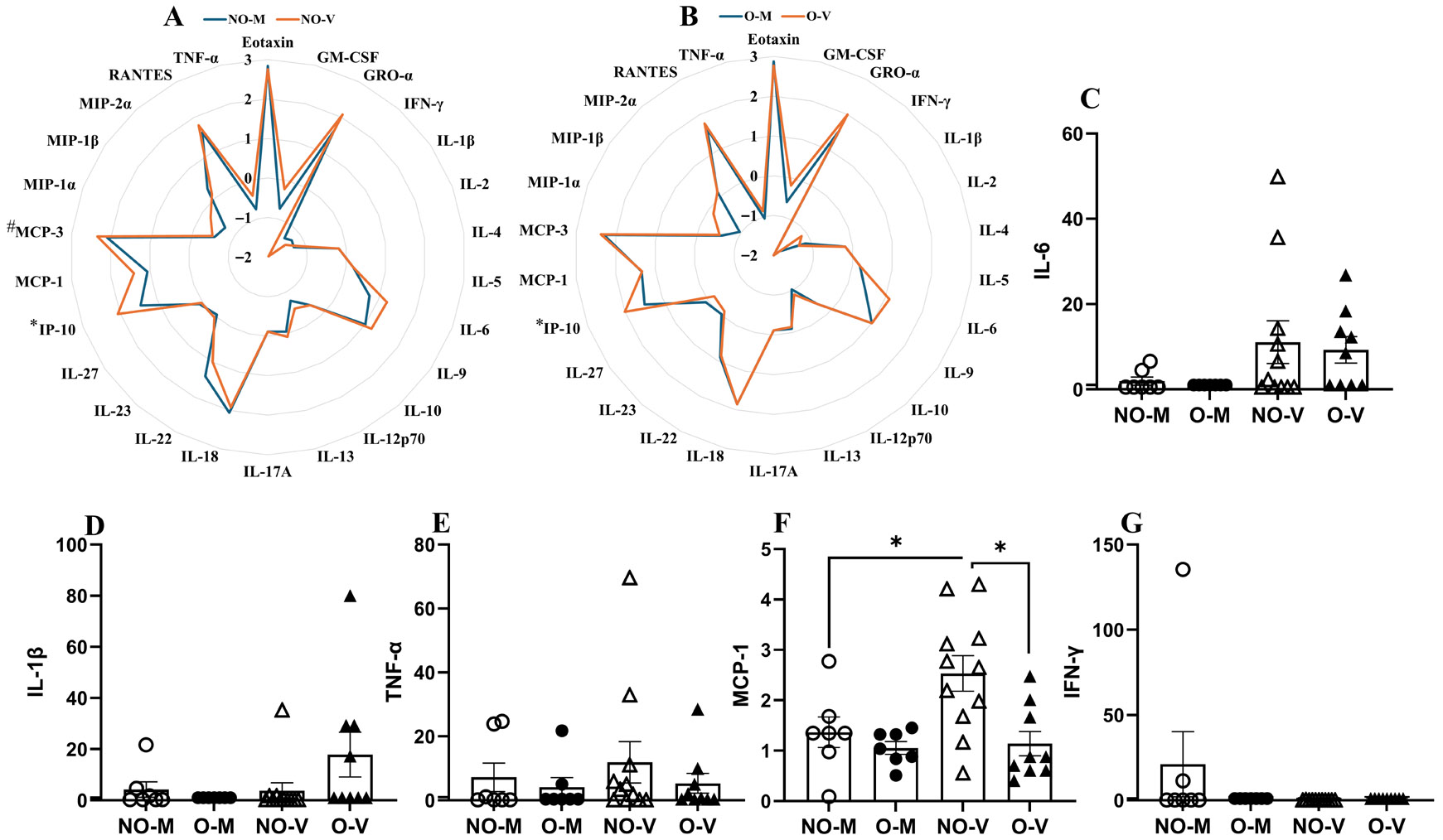
Cytokines and chemokines in plasma samples. At 3 days post-infection (dpi), medium-inoculated and virus-infected mice with or without obesity were euthanized, and 26 cytokines and chemokines were measured in the plasma samples. Comparisons of log_10_-transformed cytokine and chemokine concentrations between medium-inoculated versus virus-infected non-obese (**A**) and mice with obesity (**B**) are shown. The fold changes in concentrations of IL-6, IL-1β, TNFα, MCP-1, and IFN-γ in mice with or without obesity (relative to their respective medium controls) were then compared (**C**–**G**). Data represent mean ± standard error of mean (SEM) of 7–11 mice/group. Statistical comparison was carried out using the unpaired *t*-test with Holm–Sidak correction for multiple comparisons (**A**,**B**), and two-way ANOVA followed by Tukey’s multiple comparisons (**C**–**G**). The asterisk represents a significant difference (*p* < 0.05), and # represents a trend (0.05 ≤ *p* ≤ 0.1). NO-M, O-M, NO-V, and O-V represent the non-obese medium-inoculated, obese medium-inoculated, non-obese virus-infected, and obese virus-infected groups, respectively.

We also measured the 26 cytokines and chemokines in the plasma samples of medium and virus-inoculated non-obese and obese mice at 3 dpi. Compared to the medium-inoculated mice, virus-infected non-obese mice had significantly increased (*p* < 0.05) plasma concentrations of IP-10, and a higher trend of MCP-3 (Figure 3A). In the group with obesity, compared to the medium-inoculated, the virus-infected mice had significantly higher (*p* < 0.05) concentrations of IP-10 (Figure 3B). When fold changes in IL-6, IL-1β, TNFα, MCP-1, and IFN-γ were compared in the plasma, only MCP-1 was significantly lower in the group with obesity compared to non-obese mice (Figure 3C-G).

### 3.4. Inflammatory Changes in the Adipose Tissues

**Figure 4.**
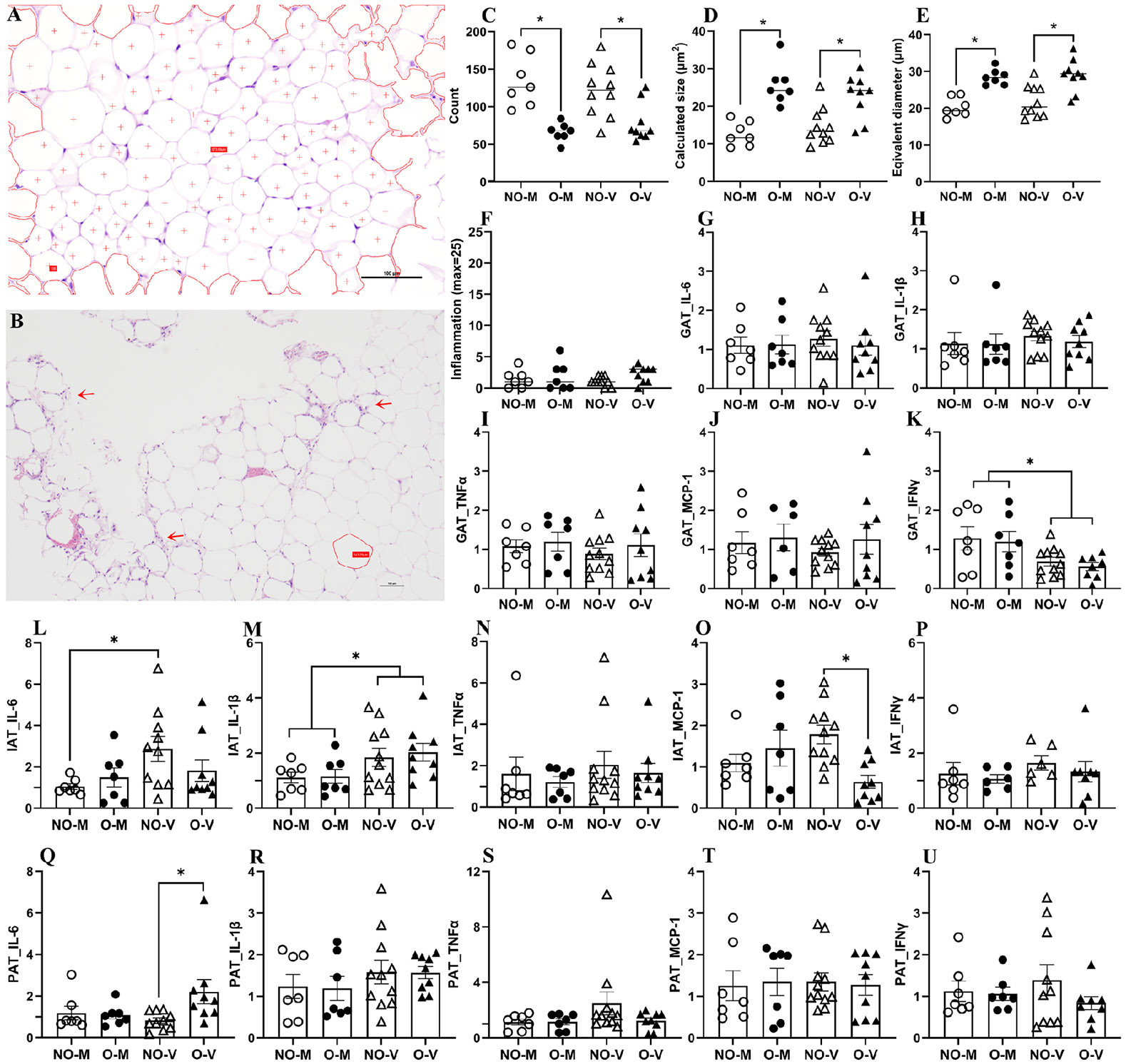
Inflammatory changes in the adipose tissues. At 3 days post-infection (dpi), various adipose tissues were collected from the medium-inoculated and virus-infected mice with or without obesity, and histopathology analysis was performed. Representative images of GATs from female mice without (**A**) and with obesity (**B**) are shown. Adipocyte count; calculated size of adipocytes; equivalent diameter of the adipocytes; and inflammation scores in histopathology in GATs are shown (**C**–**F**). Likewise, mRNA expressions of different cytokines and chemokines were determined using qRT-PCR, and fold changes for IL-6, IL-1β, TNFα, MCP-1, and IFNγ are shown for GAT (**G**–**K**). At 3 days post-infection (dpi), IAG, and PAT were also collected, fold changes for IL-6, IL-1β, TNFα, MCP-1, and IFNγ are compared in IAT (**L**–**P**) and PAT (**Q**–**U**). Data represent mean ± standard error of mean (SEM) of 6–11 mice/group. Statistical comparison was carried out using two-way ANOVA followed by Tukey’s multiple comparisons. The asterisk (*) represents a significant difference (*p* < 0.05). NO-M, O-M, NO-V, and O-V represent the non-obese medium-inoculated, obese medium-inoculated, non-obese virus-infected, and obese virus-infected groups, respectively.

To determine if IAV infection caused inflammatory changes in the GATs, GATs collected at 3 dpi were formalin-fixed and subjected to H&E staining. For the GAT, the region of interest (ROI) was first selected, covering the whole slide surface, and within the ROI, adipose cells were counted for each mouse. Representative images of a non-obese female mouse (Figure 4A) and a female mouse with obesity (Figure 4B) are shown. The adipose tissue of mice with obesity is showing scattered foci of inflammation (red arrow) and microscopic hemorrhages (Figure 4B). The number of adipocyte counts was significantly lower in mice with obesity compared to the non-obese mice, both in medium-inoculated and virus-infected mice (Figure 4C). However, the calculated size, obtained by dividing ROI by the adipocyte counts, as well as the equivalent diameter of adipocytes, were both significantly higher in the group with obesity compared with the non-obese group, irrespective of the virus-infection status (Figure 4D,E). Despite the mice with obesity showing the existence of some inflammatory changes (Figure 4B), there were no statistical differences between the medium-inoculated as well as virus-infected mice, with or without obesity (Figure 4F). We then measured the fold changes in the cytokines/chemokines in the GATs. There was no difference in the fold change in the IL-6, IL-1β, TNFα, and MCP-1 between virus-infected non-obese versus mice with obesity (Figure 4G-J). However, the IFNγ response was lower after virus infection in GAT (Figure 4K).

In IAT, IL-1β level was significantly increased following virus infection in both groups of mice; and IL-6 was significantly increased only in the non-obese mice (Figure 4L-P). Compared to the virus-infected non-obese mice, fold change in MCP-1 was significantly lower in the virus-infected mice with obesity (Figure 4O). In PAT, fold changes in IL-6 were significantly higher in virus-infected mice with obesity compared to non-obese mice, with no differences observed among other cytokines (Figure 4Q-U). Replicating viruses were not detected in adipose tissues and all values were below the limit of detection.

### 3.5. Inflammatory Changes in the Liver and the Heart

**Figure 5.**
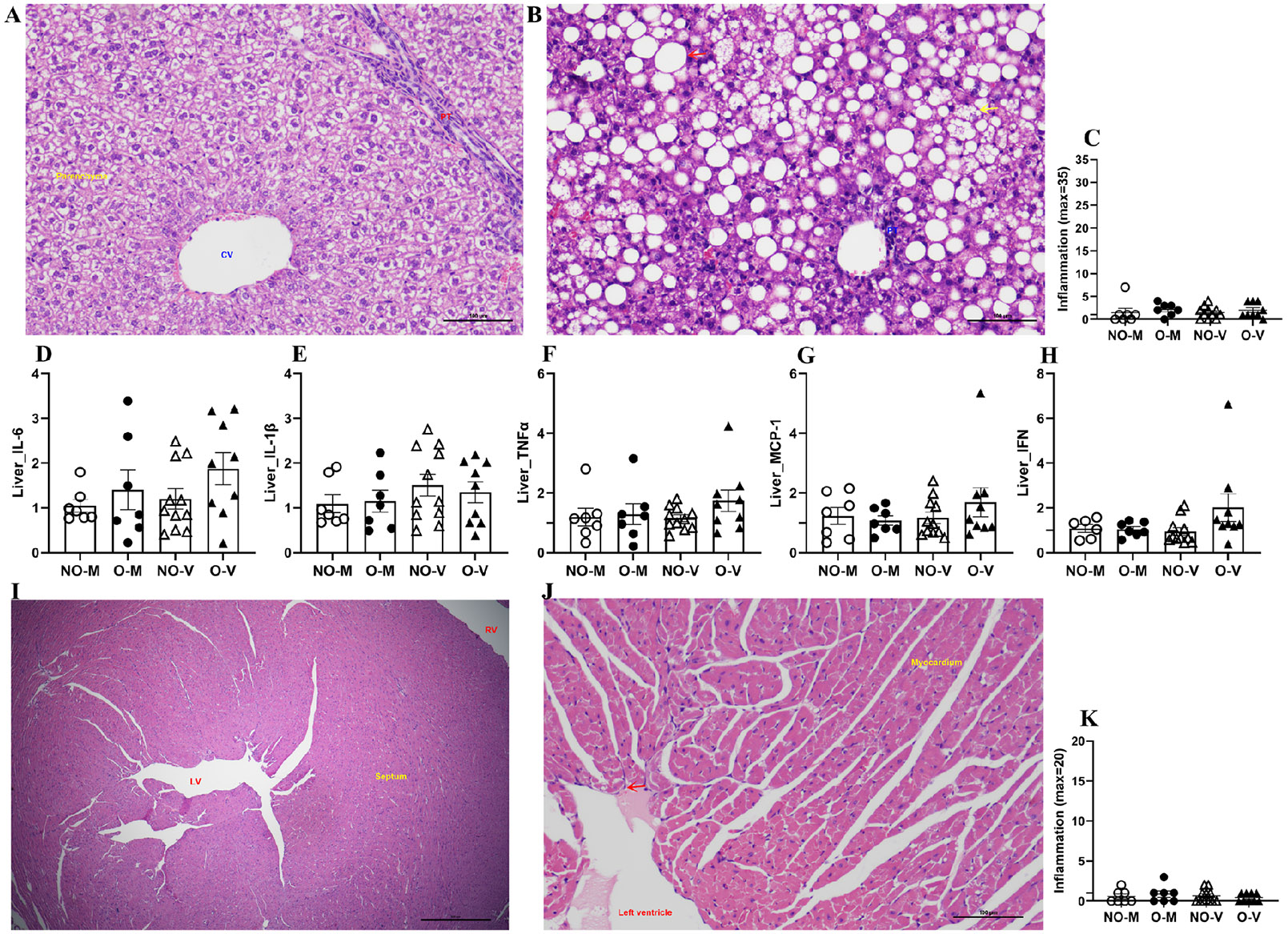
Inflammatory changes in the liver and heart. At 3 days post-infection (dpi), the liver and heart were collected. Representative images of the liver from a non-obese male (**A**) and a male with obesity (**B**) are shown, and inflammation scores are compared (**C**). The mRNA expression of different cytokines and chemokines was determined using qRT-PCR, and fold changes for IL-6, IL-1β, TNFα, MCP-1, and IFNγ are compared (**D–H**). Likewise, representative images of the heart from a non-obese male (**I**) and a male with obesity (**J**) are shown, and inflammation scores are compared (**K**). Data represent mean ± standard error of mean (SEM) of a 6–11 mice/group. Statistical comparison was carried out using two-way ANOVA followed by Tukey’s multiple comparisons. NO-M, O-M, NO-V, and O-V represent the non-obese medium-inoculated, obese medium-inoculated, non-obese virus-infected, and obese virus-infected groups, respectively.

To determine if IAV infection caused any inflammatory changes, liver samples of medium-inoculated and virus-infected mice with or without obesity were collected at 3 dpi, formalin-fixed, and subjected to H&E staining. Representative images of the liver from a medium-inoculated male without obesity (Figure 5A) and with obesity (Figure 5B) are shown. While the liver of non-obese mice is normal (Figure 5A), the liver of mice with obesity shows hepatic lipidosis that ranges from microvesicular (yellow arrow) to macrovesicular hepatic lipidosis (red arrow) with a largely unaffected portal triad (PT) (Figure 5B). In the liver, no significant changes were observed in the inflammation, by H&E staining, in IAV-infected mice compared to medium-inoculated mice, and there was no effect of obesity status (Figure 5C). There were no significant differences in the fold changes in IL-6, IL-1β, TNFα, MCP-1, and IFNγ in virus-infected mice of both groups compared to the medium-inoculated controls (Figure 5D-H). Infectious viruses were not detected in liver homogenates, and all values were below the limit of detection.

At 3 dpi, hearts were also collected, formalin-fixed, and subjected to H&E staining. Representative images of the heart from a medium-inoculated non-obese male (Figure 5I) and a virus-infected male with obesity (Figure 5J) are shown with normal structures. Even after virus infection, mice with obesity showed a normal heart with a clear ventricular lumen lined by normal endocardial endothelium (red arrow) and normal myocardium (Figure 5J). There was no significant difference in inflammatory changes by H&E staining in the heart between medium-inoculated as well as virus-infected mice with or without obesity (Figure 5K).

### 3.6. Inflammatory Changes in the Kidney and Spleen

**Figure 6.**
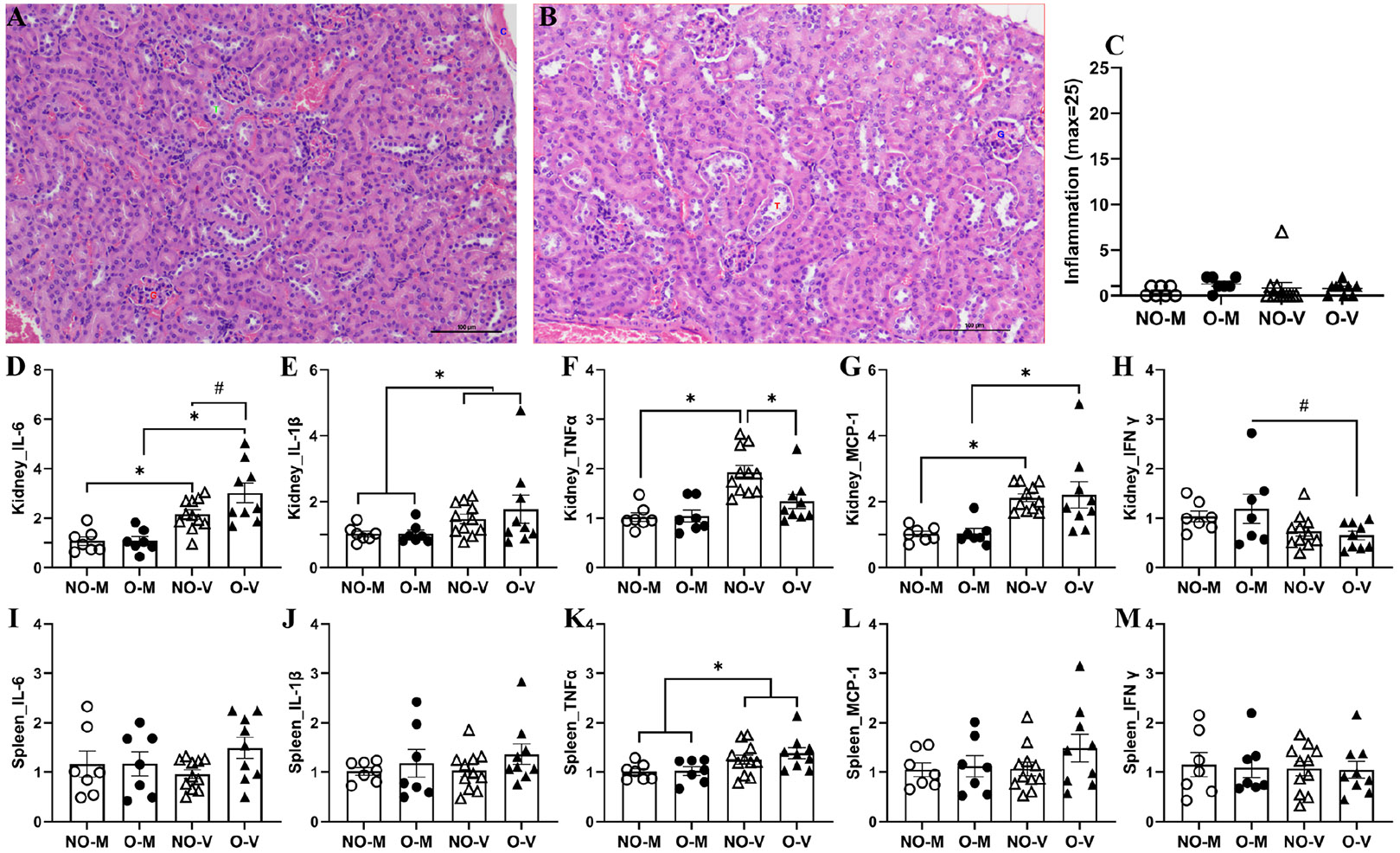
Inflammatory changes in the kidney and spleen. At 3 days post-infection (dpi), kidney and spleen samples were collected. Representative images of the kidney from a medium-inoculated non-obese female (**A**) and a virus-infected female with obesity (**B**) are shown, and inflammation scores are compared (**C**). The mRNA expression of different cytokines and chemokines was determined using qRT-PCR, and fold changes for IL-6, IL-1β, TNFα, MCP-1, and IFNγ are compared in the kidney (**D–H**) and spleen (**I–M**). Data represent mean ± standard error of mean (SEM) of 7–11 mice/group. Statistical comparison was carried out using two-way ANOVA followed by Tukey’s multiple comparisons. The asterisk represents a significant difference (*p* < 0.05) and # indicates a trend (0.05 ≤ *p* ≤ 0.1). NO-M, O-M, NO-V, and O-V represent the non-obese medium-inoculated, obese medium-inoculated, non-obese virus-infected, and obese virus-infected groups, respectively.

To determine if IAV infection caused any inflammatory changes, kidney samples from medium-inoculated and virus-infected mice were collected at 3 dpi, formalin-fixed, and subjected to H&E staining. Representative images of the kidney from a medium-inoculated non-obese female (Figure 6A) and a virus-infected female with obesity (Figure 6B) show unaffected tubules (T) and glomeruli (G). No significant changes in inflammation were observed by H&E staining between medium-inoculated and virus-infected mice with or without obesity (Figure 6C). Infectious viruses were not detected in the kidney samples of any of the mice and all values were below the limit of detection.

Significantly higher fold changes in IL-6, IL-1β, and MCP-1 were observed in the virus-infected groups compared to the medium-inoculated group (Figure 6D-H). In non-obese mice, virus infection also led to significantly increased expression of TNF-α. When comparing the two virus-infected groups, mice with obesity exhibited a trend toward higher IL-6 expression but a significantly lower level of TNFα. Additionally, a trend toward decreased IFNγ expression was observed in the virus-infected and obese mice compared to their medium-inoculated controls (Figure 6H).

In the spleen, infectious viruses were not detected in any samples either from the mice with or without obesity. Regarding the cytokines/chemokines, only the expression of TNFα was significantly increased in virus-infected compared to the medium-inoculated mice in the spleen (Figure 6I-M).

## 4. Discussion

In this study, C57BL/6J mice were fed either an HFD or an LFD for 14 weeks to induce obesity. As expected, mice with obesity displayed significantly greater body mass, increased BMI, and impaired glucose tolerance compared to the non-obese controls. These findings are in line with previous studies demonstrating similar metabolic outcomes in diet-induced obesity [30,31]. Plasma leptin and total cholesterol levels were markedly elevated in HFD-fed mice with obesity, consistent with reports linking obesity to dysregulated lipid and adipokine profiles [30,32]. In agreement with earlier works, mice with obesity also exhibited enhanced fat accumulation in multiple depots, including gonadal, inguinal, and perirenal adipose tissues, confirming the effective induction of the obese phenotype [19,30].

Histopathology analysis of lung tissues at 3 dpi revealed significant pathological changes in both groups of mice infected with the influenza virus, compared to the medium-inoculated mice. There was no difference in lung inflammation based on histopathology analysis between mice with or without obesity. Consistent with these findings, Milner et al. also did not observe significant differences in the histopathology scores between the non-obese and obese C57BL/6J mice at 4 and 8 dpi with the 2009 pandemic H1N1 IAV [33]. Likewise, following infection with the H1N1 PR8, total lung histopathology scores were comparable between non-obese and diet-induced obese mice at 5 dpi [19]. However, other observations such as numbers of lung CD45+ cells, macrophages, and neutrophils determined by flow cytometry; left lung edema as determined based on the measurement of lung weight; and fold changes in bronchoalveolar lavage (BAL) fluid protein were observed following IAV infection in mice with obesity in the previous studies highlighting increased inflammatory changes [14,19,33]. Viral titers in the lungs of mice with or without obesity at 3 dpi were comparable, indicating that obesity did not significantly alter early viral replication in this study. Prior studies in mouse models of diet-induced obesity have mostly shown comparable lung virus titers between non-obese and obese mice following infection with H1N1 PR8 or the 2009 pandemic H1N1 IAV [19,33,34]. However, in a ferret model of obesity, significantly greater virus titers were observed in the lungs at 3 dpi following infection with the 2009 pandemic H1N1 IAV, and in a mouse model a significant increase in lung virus titers was observed at 6 dpi [20]. In our study, cytokine/chemokine concentrations were comparable, yet mice with obesity exhibited increased pulmonary fold changes in IL-6, IL-1β, TNFα, but reduced fold changes in MCP-1 compared with non-obese mice. Findings from prior studies show variable results. Chandrasekaran et al. noted significantly increased concentrations of CCL20, G-CSF, and IL-6 in mice with obesity, obtained after 30 weeks of diet-treatment, following infection with H1N1 PR8 at 6 dpi while KC (CXCL1), IL-1β, and MCP-1 (CCL2) levels were numerically higher but not significant [34]. Milner et al. reported a delayed increase in CXCL1, MCP-1, and TNF-α at 8 dpi, with no differences in IFN-γ, IL-10, IL-17A, or RANTES following infection with the 2009 pandemic IAV [33].

Siegers et al. reported that LFD-fed mice had robust serum cytokine responses (IL-6, IFN-γ, IFN-α, IP-10, MCP-1, TNF-α), while 40% HFD-fed mice showed blunted responses, including lower IP-10 and delayed MCP-1 induction at 6 dpi following infection with the 2009 pandemic H1N1 strain [20]. Similarly, Cole et al. observed that systemic TNFα increased only in non-obese mice, IL-6 was delayed and IL-1β declined by 6 dpi exclusively in mice with obesity [15]. Though our study only analyzed 3 dpi following infection with a high-dose IAV, we observed that induction of MCP-1 chemokine was lower in mice with obesity compared to the non-obese mice. This is likely to be associated with the higher baseline inflammation existing in the obese compared to the non-obese mice.

Earlier studies have suggested the possibility of influenza virus replication in the thoracic adipose tissue, adjacent to the lungs, in obese mouse models [16], and detected viral RNA in epididymal white adipose tissue (eWAT) and subcutaneous adipose tissues without any difference between the obesity status of mice [13,14]. While we did not test thoracic adipose tissue in this study, the replicative or infectious viruses were not detected in GAT, IAT, and PAT of the obese as well as non-obese mice at 3 dpi. In GAT, obese mice had hypertrophic adipocytes but showed no significant histological signs of inflammation or differences in IL-6, IL-1β, or TNFα expression. Fold changes in MCP-1 trended downward, and IFNγ was significantly reduced in obese virus-infected mice. These results align with previous findings that eWAT infected with H3N2 at 7 dpi shows minimal histological changes post-infection [13] and that influenza infection using H1N1 PR8 induces significantly higher fold changes in type I IFN and IL-6 responses in non-obese but not in obese mice at 3 and 5 dpi [14]. In IAT, virus-infected non-obese mice exhibited increased IL-6, IL-1β, and MCP-1, whereas obese mice showed increased IL-1β but reduced MCP-1. Cole (2008) observed elevated mRNA transcripts for IL-1β and IL-6 in obese, but not in lean mice [15]. In PAT, obese mice showed a trend toward elevated IL-6 and decreased TNFα consistent with prior reports of depot-specific alterations in retroperitoneal adipose tissue during infection, including increased IL-1β, IL-6, and MIP-1α in obese mice [15]. These data suggest that obesity has a mild effect in altering the cytokine/chemokine responses in adipose tissues and that it occurs in a depot-specific manner.

Liver histopathology in our study revealed hepatic lipidosis in obese mice, which is also observed in prior studies of diet-induced obesity in mouse models [35,36]. However, no significant enhancement in inflammation was detected in histopathology analysis following IAV infection in both groups, with or without obesity. Although histological inflammation was absent, virus-infected mice with obesity in our study exhibited a trend toward increased hepatic expression of IL-6, TNF-α, and IFN-γ compared to non-obese mice. These results are consistent with an earlier study, which observed 6-fold increase in mRNA transcripts for TNFα and IL-6, and a 2-fold increase in mRNA levels of IL-1β in livers of IAV-infected mice with obesity at 6 dpi [15]. Replicating viruses were not detected in the liver from any of the mice. Our study showed that obesity did not alter the inflammatory changes in the heart, as determined by histopathological analysis, following IAV infection, and no replicating viruses were detected. In contrast, Siegers et al. reported cardiac alterations in HFD-fed mice infected with the A/H1N1/Auckland/1/2009 strain, including significantly increased viral loads at 6 dpi and ventricular wall thickening at 4 dpi [20].

In the kidney, virus-infected obese mice in our study showed a trend toward increased IL-6 expression and reduced TNFα levels. Short et al. observed decreased IL-6 and IL-8 expression in the kidneys at 3 dpi following infection with the 2009 pandemic H1N1 IAV infection, but in the ferret model of obesity [18]. In the spleen, obese mice infected with influenza showed elevated trends in IL-6, IL-1β, and MCP-1 compared to the virus-infected non-obese mice. A prior study in ferrets, however, observed reduced IL-6, TNFα, and IL-8 levels in the spleens of IAV-infected ferrets at 1 and 3 dpi [18], which is likely to reflect the species-specific differences in influenza pathogenesis between the mouse and ferret models of obesity.

This study has limitations such as the use of a high dose that allowed only for analysis at 3 dpi. Use of a low dose virus infection with inclusion of the acute phase (i.e., 1–3 dpi), peak disease phase (i.e., 6–10 dpi), and the recovery phase (i.e., 14–21 dpi) will provide a better understanding of the progression of inflammatory changes in extrapulmonary tissues in the future. Influenza virus responses can be strain- and subtype-specific. For example, in a non-obese BALB/c mouse model, infection with highly pathogenic influenza H7N9 could cause infection and injury in multiple organs besides the lungs [37]. Therefore, whether obesity increases virus replication and inflammatory changes in the extrapulmonary tissues following different strains and subtypes of influenza viruses, such as the emerging H5N1 avian influenza, with zoonotic potential needs further investigation. We also only focused on H&E staining, and no other inflammatory markers were tracked in extrapulmonary tissues. Regarding cytokines and chemokines, only five were compared in extrapulmonary tissues. Likewise, a larger sample size for the comparisons of cytokines/chemokines may have increased the ability to detect differences between the uninfected and infected mice with or without obesity, and to determine sex-specific differences. More elaborate studies with different infectious doses, different strains and subtypes of IAVs, multiple timepoints, and with broader considerations of inflammatory parameters are required in the future.

## 5. Conclusions

In this study, we used the C57BL/6J mouse model of diet-induced obesity to investigate whether obesity status exacerbates virus replication and inflammatory changes in the extrapulmonary tissues, such as the liver, kidney, spleen, heart, and various adipose tissues, using a high dose of 2009 pandemic H1N1 IAV at 3 dpi. Infectious viruses were not detected in any tissues other than the lungs, and obesity did not increase the IAV-infection-induced inflammatory changes in any of the extrapulmonary tissues analyzed based on histopathology analysis. The cytokine and chemokine responses, however, were altered in a tissue-specific manner between virus-infected mice with or without obesity. Taken together, our data suggest that obesity did not significantly influence extrapulmonary viral replication or pathology in this model, though minor tissue-specific immune differences were observed. Further studies in the context of different viral doses, viral strains and subtypes, different dpi’s, and with broader consideration of inflammatory parameters are necessary to continue the exploration of the effects of obesity on extrapulmonary pathology following influenza virus infection.

## Data Availability

Data presented in this study will be available upon reasonable request from the corresponding author, following the university guidelines.
